# The basic psychological needs of fitness self-testing for Chinese male college students: a qualitative perspective

**DOI:** 10.3389/fpsyg.2026.1865420

**Published:** 2026-07-06

**Authors:** Xin Zhang, Jiren Zhang, Chenhao Wu, Yucen Li, Can Zhou, Yiqiong Zhang, Xiaofen D. Hamilton, Timothy Lynch, Yongshun Wang, Mark F. Hamilton

**Affiliations:** 1 Department of Physical Education, Nanjing University of Aeronautics and Astronautics, Nanjing, China; 2 School of Physical Education, Huaqiao University, Quanzhou, China; 3 Department of Curriculum and Instruction, The University of Texas at Austin, Austin, TX, United States; 4 Department of Aviation Services and Media, Science and Technology College of Nanchang Hangkong University, Nanchang, China; 5 Department of Physical Education, Fudan University, Shanghai, China; 6 Department of Education, Yew Chung College of Early Childhood Education, Hong Kong, Hong Kong SAR, China; 7 Yew Chung International School of Chongqing, Yew Chung Yew Wah Education Network, Chongqing, China; 8 Walker Department of Mechanical Engineering, The University of Texas at Austin, Austin, TX, United States

**Keywords:** Chinese college freshmen, fitness self-tests, health-related fitness, perceptions, self-determination theory

## Abstract

**Introduction:**

Guided by Self-Determination Theory (SDT), this study examined Chinese male college freshmen’s perceptions of their fundamental psychological needs in relation to fitness self-testing. Specifically, the study focused on how the educational experience of independently monitoring and enhancing health-related fitness (HRF) supported—or challenged—the satisfaction of autonomy, competence, and relatedness.

**Methods:**

Ten participants (age: 18–19 years old) were purposively sampled from those whose fitness testing scores ranked in the top or bottom 5% of the class at the end of a semester. Each participant completed monthly self-testing sessions over 4 months. Guided by the SDT, semi-structured in-depth interviews were conducted. Constant comparative analysis was used to analyze the data to generate themes. Observation notes and student reflections were utilized for data triangulation. Peer debriefing, member checking, and negative case analysis were conducted to establish the trustworthiness of the data.

**Results:**

Four main themes emerged from the data: (a) Autonomy as empowerment and responsibility, (b) Competence satisfaction mixed with frustration, (c) Self-testing as a socially embedded practice, and (d) Self-testing as a catalyst for self-challenge, and self-regulated lifestyle development.

**Conclusion:**

The three basic psychological needs in fitness self-testing, autonomy, competence, and relatedness, were central in this context. These findings can inform future fitness self-testing protocols that support student learning, HRF, and social belonging.

## 1 Introduction

Health-related fitness (HRF), which is usually defined as the physical fitness that is directly associated with health, daily functioning, and disease prevention, and typically includes cardiovascular endurance, muscular strength and endurance, flexibility, and body composition (Keating et al., 2020), has been a common practice in schools, particularly within physical education (PE) programs worldwide (Ashley and Kawabata, 2023; Alyaqoub et al., 2024; Hamilton et al., 2025; Yang et al., 2025). Despite cross-national differences in educational philosophy, influenced by political and financial contexts, the primary purpose of health-related fitness (HRF) testing has remained consistent at its core: to assess, monitor, and enhance student fitness levels (O’Keeffe et al., 2020; Hamilton et al., 2025). Over the decades, while PE curricula have undergone substantial revisions globally (Meng et al., 2021; Tinning, 2023; Stirrup et al., 2024), HRF testing has remained a component of health and school-based PE programs in many countries (Silverman et al., 2008; Ortega et al., 2025; Yang et al., 2025). Research suggests that students and teachers can benefit from HRF testing results when they are used appropriately (Alfrey and Gard, 2019; Ashley and Kawabata, 2023; Ortega et al., 2025). As a result, HRF is one of the few health-related measures consistently evaluated and monitored in educational settings, underscoring its role in fostering lifelong health behaviors (Wang et al., 2026; Yang et al., 2025).

Higher education represents a particularly important context for promoting student HRF, as it constitutes one of the final large-scale opportunities to positively influence the well-being of a substantial proportion of the population (Zhang et al., 2024). College is a critical developmental period marked by increased autonomy and significant lifestyle changes that can shape long-term health trajectories (Abreu et al., 2023; Wang et al., 2024; Zhang et al., 2024). In China, this period is especially salient because PE is compulsory for all students during the first 2 years of university, regardless of academic major (Chen et al., 2025). In addition, all Chinese college students are required to participate in annual national fitness testing administered by PE instructors, and testing results carry high-stakes consequences, including implications for scholarship eligibility and graduation (Liu et al., 2017; Yang et al., 2025). For international readers, this institutional structure is central to understanding why fitness testing and students’ responses to it may be experienced as especially consequential during the early years of university.

It is important to note that more than half a century of widespread fitness testing has not produced corresponding improvements in student HRF (Harris et al., 2018; Alfrey, 2024). On the contrary, evidence suggests that HRF levels have stagnated or declined across educational levels, prompting scholars to question the educational and motivational value of traditional, teacher-directed fitness testing (Nolan et al., 2026; Zhang et al., 2024). Concerns have been raised regarding the extent to which conventional testing practices emphasize comparison, pressure, and performance outcomes rather than meaningful learning, autonomy, and sustained engagement in physical activity (Alfrey, 2024; Durden-Myers et al., 2025). These critiques have led to increasing interest in alternative approaches that may better support students’ motivation and long-term health behaviors (Keating et al., 2020; Alfrey, 2024). Among alternatives to traditional fitness assessment practices, fitness self-testing has gained increasing attention as an approach that promotes student self-awareness and personal accountability (Keating et al., 2020). By allowing students to assess their HRF at a time and place of their choosing, fitness self-testing encourages them to use test results to guide their own fitness improvement rather than merely reporting outcomes to teachers (Keating et al., 2020; Liu et al., 2024). This approach may be particularly relevant during the college years, a period characterized by substantial lifestyle transitions that often coincide with declines in HRF (Chen et al., 2020; Zhang et al., 2024). Moreover, by also providing direct and timely feedback on fitness levels, self-testing may help students identify strengths, address areas for improvement, and make more informed lifestyle decisions (Abreu et al., 2023; Keating et al., 2020), thereby supporting both immediate health awareness and the development of long-term physical activity habits.

To date, empirical research on fitness self-testing remains limited. Initial findings suggest that it may offer a more empowering and less pressurized alternative to traditional instructor-led assessments. For example, Graser et al. (2011) found that children enjoyed self-testing and understood its relevance to health, while Liu et al. (2024), using a Self-Determination Theory (SDT) framework, reported that self-testing supported preservice teachers’ motivation and engagement. These emerging findings highlight the relevance of SDT for examining how students experience fitness self-testing, providing a theoretical foundation for the present study.

### 1.1 SDT and basic psychological needs

SDT is a broad theory that has been used to understand individuals’ basic psychological needs for motivation and behavior change in educational settings (Ryan and Deci, 2024). At its core, SDT focuses on the satisfaction of three basic psychological needs—autonomy (i.e., feeling a sense of choice and volition), competence (i.e., feeling effective and capable), and relatedness (i.e., feeling connected to others and a sense of belonging)—and argues that individuals are more likely to engage in activities willingly and experience positive outcomes when these needs are supported (Hancox et al., 2018; Vasconcellos et al., 2020). Furthermore, Reeve and colleagues (Reeve et al., 2003) argued that internal locus of causality and volition, rather than perceived choice, are more valid indicators of self-determination. In contrast, other studies have reported that perceived choice can positively contribute to students’ motivation and psychological need satisfaction in educational and physical activity contexts (How et al., 2013; Gray et al., 2018; Hancox et al., 2018). These inconsistent findings regarding the role of perceived choice are important to the current study because our study focused on giving students control over when and how they engaged in fitness self-testing. This design allowed us to examine how perceived choice may shape students’ autonomy, motivation, and engagement within a fitness self-testing context. Of greater importance, it is well documented that social contexts such as schools play a crucial role in supporting these needs, thereby shaping students’ motivation and health behaviors (Ahmadi et al., 2023; Chiu et al., 2021; Gray et al., 2018).

Within educational contexts, SDT offers a comprehensive framework for examining the underlying motivational processes that shape teachers’ and students’ behaviors, moving beyond a focus on behavioral outcomes alone (Ryan, 2023). SDT facilitates analysis of how instructional practices, feedback, assessment, and social contexts influence distinct forms of motivation (e.g., autonomous and controlled motivation), how motivation evolves over time, and how contextual factors contribute to engagement, persistence, or disengagement (Gray et al., 2018; Hancox et al., 2018). From this perspective, SDT provides a theoretical lens for interpreting teachers’ and students’ motivation, participation, and lived experiences in educational and physical activity settings (Hancox et al., 2018; Vasconcellos et al., 2020). The theory can be used to explain why some students engage willingly and enthusiastically in learning or HRF activity, whereas others participate reluctantly or disengage, by emphasizing the role of basic psychological need satisfaction (Gray et al., 2018; Hancox et al., 2018). Specifically, SDT highlights the importance of autonomy-supportive teaching practices, opportunities for meaningful choice, competence-enhancing feedback, and supportive social relationships in fostering more self-determined forms of motivation (Ahmadi et al., 2023; Franco et al., 2025). Collectively, SDT provides a foundation for understanding how educational and HRF activity contexts can promote sustained engagement, enjoyment, and long-term well-being (Gray et al., 2018).

SDT was adopted as the theoretical framework for this study because it offers a useful lens for examining how students experience and interpret fitness self-testing within highly structured educational contexts. Within this mandatory and performance-oriented context, SDT is particularly useful for understanding how students perceive and respond to fitness self-testing in terms of their basic psychological needs for autonomy, competence, and relatedness. By focusing on these need-based experiences, SDT allows the present study to move beyond behavioral participation and examine the quality of students’ motivation and engagement, providing a theoretically grounded understanding of first-year Chinese male students’ perceptions of fitness self-testing (Keating et al., 2019). Specifically, SDT guided the design of the fitness self-testing intervention by emphasizing students’ autonomy, competence, and relatedness. For example, autonomy was supported by allowing students to decide when and where to complete the tests; competence was addressed by ensuring that students could conduct HRF self-testing with acceptable accuracy and by encouraging them to use their self-test results to first identify areas for improvement and then work toward improvement accordingly; and relatedness was promoted through opportunities to work with peers in class. SDT also informed the development of interview questions that explored students’ perceived choice, confidence in conducting fitness self-testing and improving their HRF, and interactions with peers, instructors, and TAs. During data analysis, SDT served as a sensitizing framework for coding and interpreting participants’ experiences related to autonomy, competence, and relatedness, while still allowing new themes to emerge inductively from the data.

### 1.2 Previous research on fitness self-testing and college students’ HRF

Research with college students consistently demonstrates that satisfaction of these psychological needs promotes positive behavior change (Dempsey et al., 2025; Grao-Cruces et al., 2020; Pettersson et al., 2021; Taylor and MacLeod, 2024). Autonomy has been linked to greater engagement in exercise and self-directed behaviors, competence to persistence and confidence in fitness management, and relatedness to peer support and collaborative learning (Abdulla et al., 2022). Importantly, the satisfaction of all three needs together is considered essential for sustainable behavior change (Quartiroli and Maeda, 2014), though the relative importance of each need may vary across contexts (Pettersson et al., 2021).

Previous research has also indicated that first-year college students are at a critical developmental and transitional stage characterized by significant lifestyle changes that may have lasting health consequences (Gropper et al., 2020; Winpenny et al., 2020). The transition from highly structured secondary education to a more autonomous university environment often leads to reduced HRF (Zhang et al., 2024) and disruptions to previously established exercise routines (Brown et al., 2024; Gutiérrez-Espinoza et al., 2025), suggesting that regular participation in fitness-related activities may not yet be firmly established at university entry. Consequently, the first year of college represents a critical window for intervening in students’ fitness-related behaviors. Moreover, evidence indicates a higher prevalence of overweight and obesity among Chinese male college students compared to their female peers (Beaudry et al., 2019; Chen et al., 2020), which may further compromise HRF, putting them at a higher risk level for poor health. This concern is underscored by findings showing that students with higher body mass index (BMI) at the beginning of college are less likely to improve their aerobic fitness and muscular strength and endurance over time (Zhang et al., 2024). Focusing on first-year male students allows researchers to identify early fitness-related challenges, examine behavioral and environmental influences, and inform timely interventions aimed at promoting physical activity, improving health-related fitness, and fostering lifelong healthy habits during this pivotal transitional period. However, it is important to clarify that the study focused on male students not because biological sex alone determines HRF risk, but because prior evidence has identified a specific risk pattern among male college students, including higher overweight and obesity prevalence and lower performance in certain HRF components.

## 2 Purpose of the study

Given the pressing need to promote HRF in higher education and growing concerns about the limitations of teacher-directed assessments, this study explores fitness self-testing as a potential alternative. Specifically, guided by SDT, the study examines how first-year Chinese male college students perceive fitness self-testing in relation to autonomy, competence, and relatedness. The findings aim to provide insights into how fitness self-testing may support motivational processes, promote lifelong health behaviors, and address the ongoing decline in HRF among college students.

## 3 Methodology

The study was approved by the IRB committee of the senior author’s university before any data were collected. Participants signed the written consent form before taking part in the study. No personal information was used at any time.

### 3.1 Sampling and participants

Participants were selected using purposeful sampling, as described by Patton (2015), to capture a diverse range of information-rich accounts of fitness self-testing experiences. Specifically, extreme case sampling, a form of purposeful sampling (Palinkas et al., 2015; Ryu et al., 2025), was employed to identify cases at the outer ends of the distribution on a parameter of interest, thereby enabling a deeper and more nuanced understanding of the range of students’ experiences with fitness self-testing. This approach is consistent with qualitative research aims to generate analytic insight by examining cases that most clearly reveal underlying processes and experiential contrasts (Ryu et al., 2025).

The participants (*n* = 10) were first year male college students, aged 18 to 19 years due to the use of the standardized schooling system in China. The sample size was deemed sufficient because qualitative research literature suggests that relatively small samples can achieve adequate depth and saturation when the goal is to generate rich, in-depth understanding (Roberts et al., 2026). Specifically, Sandelowski (1995) argued that sample sizes of 6 to 10 participants may be appropriate for qualitative inquiry when they allow researchers to obtain detailed data and reach thematic saturation. The participants’ HRF test results, which were tested by their PE instructor and teaching assistants (TAs), were the top and bottom five in a single-sex required PE class consisting of 49 students at the end of the semester. All participants resided on campus without a part-time job, which is typical for all Chinese first-year college students.

### 3.2 Settings

Participants were enrolled in a required PE class which met once per week for 90 min over 16 weeks. The class content consisted of multiple sport activities such as volleyball, basketball, and fitness related physical activities. The PE instructor taught PE classes for 16 years while the two TAs were graduate students in the physical education teacher education program at the same university.

As noted earlier, college students in China are required to participate in annual mandatory fitness testing, even though formal physical education coursework is only required during the first 2 years of college (Keating et al., 2019; Zhang et al., 2024). All fitness testing items are administered by trained physical education instructors. During the first 2 years, students are typically tested by their own physical education instructors, as they are still enrolled in required PE courses. Junior and senior students, in contrast, are usually tested by a designated group of physical education instructors at the institutional level. In accordance with China’s Ministry of Education’s requirements, fitness testing is commonly conducted early in the academic year—often during the fall semester (around October or November) for incoming students and, in some cases, during the spring semester for upperclassmen—although specific timing may vary by university. Importantly, fitness testing results are used to determine eligibility for academic scholarships, and passing the required fitness assessments is one of the conditions for college graduation (Liu et al., 2017).

As a part of the course requirements in the current study, participants took part in monthly fitness self-testing sessions. Unlike teacher-centered fitness testing, which requires students to be tested by their instructors only during scheduled class sessions once per academic year, self-testing allows participants to use the mobile app — “Jumping Rope Every Day (Li et al., 2025; Wei et al., 2025) — to take HRF self-tests in a private setting of their choice, as many times as they liked, with a minimum of once per month. The app verifies student performance through video recording and AI-based motion recognition (Wang et al., 2026). It supports the following tests: one-mile run, pull-ups, sit-and-reach, and VO_2_ max; BMI was manually entered by participants. The app was endorsed by the Chinese government for self-testing to ensure the validity of the results (Diao and Ma, 2025; Wang et al., 2026). A previously published study has validated its results against instructor-administered tests (Wang et al., 2026).

The highest score was recorded as their testing results at the end of the semester regardless of the number of self-testing attempts completed. The following test items, which are the required testing items included in the nationwide youth fitness testing battery developed by the Chinese government (Yang et al., 2025), were used for each self-testing: body mass index (BMI), an index calculated from height and weight, as an indirect and rough index related to body fat percentage, one-mile run and VO_2_ max for cardiovascular endurance, which was assessed using a standardized VO_2_ max tester, with the highest value from three trials recorded, sit-and-reach for flexibility, and pull-ups for upper body strength. Abdominal muscular strength and endurance were not tested because this item is not required for male students in China (Yang et al., 2025). These testing items are almost identical to the testing items used in educational settings around the world (O’Keeffe et al., 2021; Stephenson et al., 2022; Harte et al., 2025). Instead of using a pass/fail evaluation method similar to those used in the US and European Union (EU), however, the Chinese fitness test battery used a 100-point scoring system (i.e., the sum of all the testing items) with four categories: fail (i.e., below 60 points), pass (i.e., 60–79), good (i.e., 80–89), and excellent (i.e., 90 or above) and the BMI test does not assign a failing score, regardless of the BMI value (Yang et al., 2025). The participants were instructed to work on their improvement on all the testing items if they were not within the excellent category. No competitions among peers were encouraged.

Participants were told that they could use the mobile app, “Jumping Rope Every Day” (Li et al., 2025; Wang et al., 2026) to self-test their HRF. The mobile app video recorded the testing process and results, except for the BMI, which were manually entered by the participants using their height and weight values. The PE instructor could check who and when students had taken the tests using the app. In addition, the PE instructor left the scales for measuring weight and height in his office, and they could use them before or after their class each week or by appointment. The app also automatically provided the participants with specific HRF exercise feedback based on their testing results in comparison with the cut-off values. The PE instructor and the two TAs tested the participants’ HRF at the end of the semester. The sum of the four testing items was used to rank students’ HRF for the purposeful sampling. The AI-supported mobile app automatically generated individualized, instant feedback after each self-test. The app first compared participants’ real-time test results with national college student fitness norms and then analyzed their performance strengths and weaknesses across BMI, one-mile run, sit-and-reach, and pull-ups. Based on each participant’s weaker fitness components, the app provided targeted exercise suggestions, including recommended training frequency, intensity, and practical fitness improvement plans. In this way, the app created a complete closed-loop process of self-testing, results-based feedback, and exercise guidance. Participants were able to understand the AI-generated feedback and recognized its alignment with official fitness evaluation standards. The PE instructor and TAs also encouraged students to use the AI-generated feedback to improve their HRF. However, no data were available to indicate whether participants trusted the AI-supported feedback.

Three of the researchers served in dual roles as investigators and as the instructor and TAs in this study. Accordingly, particular attention was given to how these relationships with participants may have influenced the research process. We believe that the rapport established with the class facilitated candid communication regarding fitness self-testing. Furthermore, the researchers’ instructional roles enhanced the fidelity of the study, as they reflected the authentic dynamics instructors commonly encounter when navigating relationships within the instructional hierarchy (Luguetti et al., 2025).

Although the rapport the researchers developed with participants helped mitigate potential concerns, we also instituted explicit safeguards to minimize any perception of coercion and to reduce social desirability bias (Patton, 2015). Because one of the researchers was the course instructor of record, it was essential to create structures that ensured student participation remained entirely voluntary and free from undue influence. To this end, all recruitment and consent procedures were conducted by the two TAs in the instructor’s absence. The TAs also assumed full responsibility for data collection, which prevented the instructor from knowing which students agreed to participate. This separation was critical for protecting student autonomy and for maintaining the integrity of the consent process. Students were explicitly informed that their decision to participate or decline participation would not affect their course grades, standing, or instructor evaluations, as stated in the IRB-approved informed consent form.

### 3.3 Data collection

Individual semi-structured interviews were conducted in Chinese to generate in-depth qualitative data. The interview questions were guided by SDT and focused on participants’ satisfaction of fundamental psychological needs in the context of fitness self-testing. Specifically, the questions addressed: (a) perceived autonomy, including freedom of choice in conducting self-tests (e.g., What is your opinion on the freedom to choose the testing time, place, and testing items? How much control did you have for fitness self-testing?); (b) perceived competence in carrying out the tests (e.g., How confident did you feel when self-testing your HRF? What problems did you encounter when self-testing your own HRF?); and (c) relatedness, or sense of belonging associated with the testing process (e.g., What relationships with your PE instructor and TAs, peers, parents and other relatives, and friends, if any, emerged through the self-testing process? How, if at all, did self-testing influence your existing relationships with your PE instructor and TAs, peers, parents and other relatives, and friends?). A summarized interview guide is presented in Table 1. Each interview was conducted in a private room and lasted approximately 45–60 min. At the end of each question, the interviewer provided a summary of the participant’s comments to confirm understanding, allowing participants to clarify their perspectives, and enabling researchers to identify key issues. Each participant was interviewed once, resulting in a total of 10 completed interviews. All interviews were digitally recorded.

**Table 1:** Interview questions.

Main theme	Interview questions
Autonomy	1. In fitness self-testing, what aspects (time, location, frequency, test arrangement) did you feel you could control freely?
	2. What problems or difficulties did you encounter in scheduling your own self-tests?
	3. How did the freedom of self-testing affect your daily study and life arrangements?
Competence	1. How confident were you in accurately completing fitness self-tests?
	2. What challenges have you faced during self-testing (e.g., app operation, venue arrangement, weather, physical state?)
	3. After receiving your self-test results and AI-generated feedback, how did you determine what strategies to use to effectively improve your HRF?
	4. What kind of help do you hope to receive to better improve your fitness based on test results?
Relatedness	1. How did your classmates or friends influence your experience with fitness self-testing?
	2. What activities did you usually do together with peers regarding self-testing and physical exercise?
	3. How did the instructor or TAs support your fitness self-testing?
	4. What relationships, if any, did you develop with peers, friends, your physical education teacher, or TAs through the fitness self-testing process?
Overall evaluation	1. What else do you want to share with us concerning your overall experience associated with the monthly fitness self-testing?
	2. What suggestions do you have for optimizing college fitness self-testing arrangements and content?

Observations were conducted throughout the semester by one of the TAs, who was a non-participant observer, even though the entire fitness self-testing process was not observable during the PE classes each month as participants usually conducted the tests outside of class. The observation notes related to the research were documented only by the TA, who was not involved in grading. The instructor did not have access to research observation data until after final grades were submitted. This separation maintained confidentiality and reduced potential coercion. The participants’ questions, or comments on their own fitness self-testing were discussed in the classes, adding the benefit of promoting reflections and deeper learning (Bjørke and Quennerstedt, 2026). Moreover, non-participant observations were carried out in natural instructional settings to gain contextualized insight into students’ behaviors, interactions, and engagement related to fitness self-testing. Detailed field notes documented instructional practices, peer interactions, and students’ verbal and nonverbal responses. Field notes focused on patterns of interactions, expressions of engagement or disengagement, questions related to self-testing, and contextual features shaping the testing experience. Given the cultural context in China, where students usually demonstrate limited verbal participation in class, observational attention extended beyond spoken interactions. Guided by the SDT framework, particular attention was paid to nonverbal behaviors such as body posture, gestures, facial expressions, eye contact, and attentiveness as indicators of students’ experiences of autonomy, competence, and relatedness. An example was that some students did not make direct eye contact and displayed slight nervous body movements when discussing how to improve their HRF with their peers in class. These observations suggest that students may have experienced challenges in improving their HRF, even when they did not verbally express those difficulties.

Observational data were used to complement and contextualize interview findings, supporting a more comprehensive understanding of students’ motivational experiences with fitness self-testing. Specifically, students’ interview comments about increased responsibility for improving their own HRF were supported by observations of students checking their self-testing results after each test, asking the instructor or teaching assistants how to improve specific fitness components when they received low scores, and discussing strategies for improvement. Participants’ frustration with their inability to improve their pull-up scores was reflected in their informal comments about how difficult it was to see improvement on the pull-up test. Similarly, interview findings related to peer support were supported by observations of students making plans to exercise together and discussing their physical activity progress in class.

### 3.4 Data analysis and trustworthiness

The interviews were conducted in Mandarin Chinese and audio recorded. All recordings were transcribed verbatim in Chinese by the research team. Data analysis was conducted using the Chinese transcripts. Because the interviews were conducted in Mandarin Chinese, analyzing the original Chinese transcripts allowed the research team to preserve participants’ intended meanings, cultural nuances, and context-specific expressions during coding. However, the research team also recognized the potential risk that findings translated into English may not fully capture all linguistic and cultural meanings. To reduce this risk, bilingual researchers conducted translation and cultural contextualization, the corresponding author verified the English transcripts against the original Chinese recordings, and back-translation was used to enhance translation accuracy and trustworthiness. Specifically, data were analyzed and coded according to commonalities and differences described around their fitness self-testing as informed by the SDT. The analysis combined inductive (data-driven) (Thomas, 2006) and deductive (theory-driven) approach (Alyaqoub et al., 2024). This combination was chosen because the inductive approach allows for the identification of patterns and the development of abstract insights that generate new knowledge such as the theme 4, while the deductive approach applies existing theories to explore prior knowledge within a different context (Wrench and Garrett, 2008; Alyaqoub et al., 2024).

The primary analytic method was reflexive thematic analysis (Nowell et al., 2017). Within this approach, we employed constant comparative techniques (Richards et al., 2023) were used to systematically compare data extracts across participants and time points to deepen familiarization and support theme development. In this study, “constant comparison” refers to an analytic strategy used to refine codes and identify patterns within reflexive thematic analysis, rather than a separate methodological commitment. Interview transcripts and observational field notes were analyzed iteratively and concurrently to ensure alignment with the data collection process. First, three researchers independently read the interview transcripts multiple times to become familiar with the data. The research team engaged in ongoing self-reflection throughout this phase and the entire analytic process. Initial impressions and potential biases were documented to ensure that prior knowledge of SDT did not overshadow participants’ perspectives.

Second, three coders independently assigned initial codes to relevant text segments. The coders then compared and discussed codes related to autonomy, competence, and relatedness to develop an initial coding framework. At the same time, the coding was also data-driven, allowing themes beyond SDT to emerge. Codes were compared and discussed, resulting in the development of an initial coding framework. Observational data were analyzed alongside interview data as a complementary source of qualitative evidence, consistent with the multi-method data collection strategy. Field notes were first subjected to open coding to identify salient behaviors, interactions, and contextual features related to fitness self-testing. These codes were then refined through focused coding, with attention to patterns of engagement, peer interaction, and verbal and nonverbal communication during class activities. Guided by SDT, observational codes were examined in relation to indicators of autonomy, competence, and relatedness. To enhance trustworthiness (Smith and McGannon, 2018), researcher triangulation was employed. Multiple members of the research team independently reviewed interview transcripts and field notes and engaged in regular discussions to compare interpretations and resolve discrepancies. Observational findings were used to corroborate, elaborate on, or complicate interpretations derived from interview data. For example, the theme 4 was carefully examined against observational data, as it was not predicted by our initial theoretical framework and required additional verification. This iterative and collaborative process strengthened the credibility of the analysis and supported a more nuanced understanding of students’ motivational experiences with fitness self-testing.

Third, the constant comparative method was applied within each structural code (Richards et al., 2023). New data extracts were systematically compared with previously coded data to identify similarities, differences, and contradictions, leading to the construction of initial themes. Fourth, peer debriefing was conducted, during which peers independently reviewed the themes and supporting evidence while consulting the full dataset. Divergent interpretations were discussed until consensus was reached. Finally, the research team met to refine and finalize themes and subthemes.

Negative cases (e.g., testing scores were not improved at the end of the semester; there were no added interactions with the PE instructor and TAs because of the self-testing) were defined as data segments that complicated or challenged emerging themes. These cases were identified through constant comparison across participants and time points and were used to refine theme development, clarify the boundaries of each theme, and ensure that the analysis captured variation as well as dominant patterns in participants’ experiences. For instance, although most participants reported that fitness self-testing helped them make new friends and develop stronger peer connections, a few participants stated that they did not experience this form of relatedness because they preferred to work independently. This negative case prompted the research team to refine the relatedness theme by considering the specific conditions under which fitness self-testing may promote social connection. Rather than assuming that self-testing automatically enhances relatedness, the analysis suggests that peer interaction, shared goals, and supportive class structures may be necessary for relatedness to emerge. The final themes and subthemes were then returned to participants for member checking to further establish credibility and trustworthiness.

### 3.5 Positionalities of researchers

The research team’s positionality shaped the design, data collection, and interpretation of this study. Several members of the research team had prior experience conducting research on fitness testing, fitness education, and physical education pedagogy, which informed our interest in examining fitness self-testing as an alternative to traditional teacher-directed fitness assessment. This background allowed us to design a study that attended not only to students’ fitness outcomes but also to their motivational experiences during self-testing.

Self-Determination Theory (SDT) also shaped our methodological decisions. Because we approached fitness self-testing through the lens of autonomy, competence, and relatedness, the intervention was designed to provide students with opportunities to choose when and where to complete self-tests, use their results to identify areas for improvement, and interact with peers during the process. These theoretical commitments also informed the interview guide. For example, students were asked about their perceived choice in completing self-tests, their confidence in conducting the tests and improving their health-related fitness, and their interactions with peers, instructors, and teaching assistants.

During data analysis, our familiarity with SDT sensitized us to participants’ experiences related to autonomy, competence, and relatedness. At the same time, we recognized the risk of overinterpreting the data through the theory. To reduce this risk, we used a hybrid inductive–deductive approach, allowing codes and themes to emerge from participants’ accounts while also examining how these patterns related to SDT. The research team engaged in ongoing discussions, peer debriefing, and comparison of data across participants to ensure that the final themes reflected participants’ experiences rather than only our theoretical assumptions. Negative cases were also examined to refine the interpretation, such as instances in which some students preferred to complete self-testing independently and did not experience increased peer relatedness.

The research team’s cultural and linguistic background also influenced data interpretation. Because the interviews were conducted in Mandarin Chinese and most members of the research team were familiar with Chinese educational and cultural contexts, we were able to attend to culturally specific meanings in participants’ descriptions of fitness testing, peer interaction, and help-seeking. At the same time, we remained aware that our shared cultural knowledge could shape interpretation. Therefore, we used team discussions, transcript verification, and translation checks to support reflexivity and enhance the trustworthiness of the findings.

## 4 Results

### 4.1 Overview

Interpreted through SDT, the data from the current study showed that fitness self-testing supported motivation by addressing autonomy, competence, and relatedness. Self-testing gave students greater ownership over their fitness behaviors, helped them recognize both their confidence and challenges in improving HRF, and created opportunities for peer support. Unlike teacher-directed assessments that of emphasized comparison, self-testing shifted students’ focus toward self-improvement and competing against their own previous performance. Overall, fitness self-testing created a motivational climate that helped students view fitness behaviors as more personally meaningful and sustainable. Four themes emerged and the following sections present detailed accounts of these themes. To ensure clarity and confidentiality, participants are identified as Participant 1 (P1), Participant 2 (P2), and so forth, through Participant 10 (P10).

### 4.2 Theme 1: autonomy as empowerment and responsibility

This theme was evident among all participants, suggesting that the participants felt that empowerment came along with responsibility. The two subthemes were: (a1) Perceived control power and flexibility with reduced pressure, and (a2) Self-discipline centered on competing with oneself rather than external comparison.

#### 4.2.1 (a1) perceived control power and flexibility with reduced pressure

All participants welcomed the flexibility in taking the tests at a time and place suitable for them and as many times as they wanted. P6 stated “I really did not pay attention to my HRF before. I do now, because my PE instructor says that I oversee improving my HRF. It becomes my job now.” P1 said:

I think it is good to be able to test myself at the time and place that I like. The added option makes me feel much less stressed out like before as there was only one chance to take the tests. Once I know that I can try multiple times to get the best results, I feel relaxed and began to have fun while doing it. (P1)

P1’s statement reflected the reduced pressure he experienced because of having the freedom to choose.

The flexibility embedded in fitness self-testing allows me to choose the best weather and the best body condition to take my tests (we sometimes took the tests when it was raining). I took the tests whenever I feel I am ready and was not forced by my PE instructor. Self-testing fits college students better because it helps us learn how to take responsibility for our own lives. It (self-testing) is a good practice. (P5)

#### 4.2.2 (a2) self-discipline centered on competing with oneself rather than external comparison

Despite the participants’ reaction to the empowerment brought by the self-testing, they also realized that self-discipline played an essential role in implementing fitness self-testing. P1 pointed out that “I had to plan my time well as my PE instructor did not plan the fitness testing for me.” P7 echoed this: “it (self-testing) is a good way to practice self-discipline as I need to find time for it. As you know, my PE instructor told me what to do before and I do not need to worry about it.” P2 further noted:

I definitely need to keep exercising and testing repeatedly to get better results. This required a lot of self-discipline as everything is on myself. If I am not satisfied with my score, I must keep working on it by myself.

I really worked to improve my own scores this semester and had to discipline myself to find time and space to enhance my HRF. It was not easy, as sometimes I wanted to go out with my friends for fun instead of exercising. However, I kept reminding myself that I needed to work out regularly because my test scores would improve only if I put in consistent effort. This was my first time experiencing this kind of self-discipline. (P9)

Besides self-discipline, participants also noted competing against themselves. Although peer competition was strongly discouraged in class, participants pointed out that the self-testing made them compete against themselves.

It (fitness testing) was a one-time deal before; I was not bothered by it as my PE teachers never asked me to compare my new scores to the old ones. I simply just did it to complete one task. Now I need to see the improvement in my HRF each time, I must compare my testing results each time. Because of the comparisons each time, I am motivated to beat my previous records. Competing against myself is interesting and I like to challenge myself this way. (P4)

My previous PE instructors never asked me to compare my current fitness test results with previous ones, even though I took the tests every year. They only cared about whether I passed. With the monthly self-testing, I feel more responsible for improving my HRF. I feel like I’m in control, but at the same time, I also realize that I have more responsibility because it is now entirely up to me. So, it was my first time to learn how to be responsible for my HRF. It is hard to be honest with you: I was not used to taking on such an important responsibility. (P6)

### 4.3 Theme 2: competence satisfaction mixed with frustration

Participants described both competence satisfaction and competence frustration in relation to fitness self-testing. On the one hand, they expressed confidence in their ability to accurately conduct the self-tests and report the result using the app, indicating a sense of competence in managing the testing procedures themselves. On the other hand, participants experienced competence frustration when attempting to translate test results into meaningful improvements in their HRF. Although immediate feedback was provided following each testing session, many participants described uncertainty and difficulty translating this information into meaningful HRF improvement, reflecting a perceived gap between testing competence and behavior change. P3 pointed out: ‘I feel very bad because my self-testing score on pull-ups was always the same. I see little differences. I do not know how to improve it. This testing item is too hard for me.’ ‘I just do not know how to improve my HRF and felt very sad when I saw the same testing scores over and over again (P7).’

I know what I need to work on (my HRF) because of the self-testing each month. I am very disappointed with my poor scores as I do not know how to enhance my performance. Sigh, it is very hard on me, I am very frustrated with it. (P4)

I think that I know how to do the self-testing now. Because of the monthly testing, I understand which areas of my HRF need improvement, which gives me more awareness than before. However, I still feel unsure about how to improve those areas. For example, I always scored zero on the pull-up test. I want to improve my score, but I don’t feel confident about how to do that, even though the AI feedback suggested more strength training. (P9)

### 4.4 Theme 3: self-testing as a socially embedded practice (relatedness)

Although fitness self-testing is formally positioned as an individual activity, participants revealed that it was often experienced and enacted as a socially embedded practice. Rather than approaching self-testing in isolation, participants described actively organizing the process around relationships with peers, suggesting that participation was shaped by social connection rather than individual obligation. Given that all participants reported that their relationships with the instructor and TAs did not change because of self-testing, relatedness appeared to develop primarily through peer interactions rather than through instructor or TA relationships. The absence of interaction with PE instructors, teaching assistants, or parents suggests that the social benefits of self-testing were primarily confined to peer networks, highlighting a limited recognition of relational needs beyond peer relationships among the participants. The two subthemes were: (c1) shared preparation and mutual support, and (c2) alignment of commitment and capability.

#### 4.4.1 (c1) shared preparation and mutual support

Fitness self-testing provided opportunities for participants to engage in collaborative activities, thereby strengthening their social connections. P1 noted;

I always take it (fitness self-testing) with friends, and even before the tests, I’ll ask people I’m close to practice together. It’s like when you ask your classmates to study with you before an exam; this kind of thing has become a part of strengthening our social connections.

P10 similarly shared,

The friends I usually exercise with tend to pay as much attention to fitness self-tests as I do. We all care quite a bit about the results. We run together regularly, play ball occasionally, and hit the gym too.When I go for it (fitness self-testing) with friends, I feel our relationships get better. Plus, taking tests and working out together is way more fun than doing it all alone. (P10)

The comments made by P1 and P10 suggested that preparing for fitness self-testing with peers functioned similarly to collaborative academic preparation: it provided social support, shared accountability, and encouragement, which strengthened students’ sense of relatedness and helped sustain their engagement in fitness activities.

In addition, P6 mentioned that he and his peers developed stronger bonds through exercising together on a regular basis. However, other participants did not provide similarly specific evidence regarding the relatedness and behavioral change.

I often ask my roommates or classmates to join me for fitness self-tests, ball games, workouts, or gym sessions. We keep this up nearly five days a week. Right after the self-testing, we make it a point to go for an evening run together, and those runs are really helping me improve my HRF. Besides that, after classes, we’ll head to the gym for extra exercises, then grab a meal. Going through all this with them, especially pushing through the fitness self-testing, keeps me motivated. (P6)

It is important to note that relatedness could also be developed through competition, as indicated by P8, even though competition was not encouraged in class because it may be harmful if overemphasized.

My classmates and I put a lot of effort into focusing on it (fitness self-tests). Sometimes, we even end up comparing with each other. Sometimes it’s about who gets better results, and sometimes it’s about who performs more evenly across all items.

#### 4.4.2 (c2) shared needs, stronger bonds

This theme illustrates how relatedness was developed through students’ shared needs and goals for improving their specific components of HRF. In other words, fitness self-testing served as a bridge for forming new social connections, as participants encountered peers with similar needs for improving their HRF. For example, participants who needed to improve their pull-up scores tended to work together on upper-body strength training, while those who needed to improve their aerobic fitness grouped together because they shared similar goals. These interactions often evolved into ongoing fitness partnerships, through which students supported one another, exchanged improvement strategies, and sustained their engagement in physical activity. P9 mentioned;

I’ve met a ton of different friends through doing fitness self-testing, they’re from different colleges and classes. Like, if someone’s really good at a certain event and I’m not, they’ll help me out. During this, we’ll chat about tips and stuff to improve our test scores. These new friends, we’re closer than just classmates.

Other participants further supported this experience:

It is surprising that I made new friends because of my poor fitness self-testing scores. I realized that I needed to improve my HRF, which I had not cared about before. Because we struggled with aerobic fitness and disliked running, we joined the basketball team together this semester, which led to shorter one-mile run times. We now continue to play basketball regularly, even when fitness self-testing is no longer required. It is not about fitness anymore, it is about friendship and having fun. (P3)

I make plans with my classmates to take it (fitness self-test) at night. For one thing, I feel more relaxed both physically and mentally in the evening, so the test goes a lot easier. For another, it’s a good way to build friendships with friends. Since we take tests like the running one together at night, I end up meeting new people who I can run with regularly. (P7)

### 4.5 Theme 4: self-testing as a catalyst for self-challenge, and self-regulated lifestyle development

Beyond satisfying the three basic psychological needs outlined in SDT, participants also described the emergence of new internally driven motivational forces that deepened their commitment to fitness behaviors. These motivations reflected a process of internalization, where students began to engage in fitness self-testing not only to meet external requirements but also for personal growth and self-actualization. P8 explained,

I think doing it (fitness self-testing) serves two purposes. One is to check if I’m keeping myself in pretty good physical shape. The other is that I want to challenge myself, and for that, I’ll just try my best to push myself, to take on that challenge. I prefer to taking self-testing; I just focus on beating my last score, trying to outdo myself.

Other participants also echoed this:

I set small goals for each test. For example, if I ran one mile in 12 minutes last month, I aim for 10 minutes this month. Since I can take fitness self-testing many times, I can improve gradually. That way, things will get better for me little by little, my scores will improve step by step, and in the end, both my test goals and results will keep inching up. (P1)

I think applying this (fitness self-testing) method to study and daily life could be really helpful. Like, take working out for example. After testing, you make plans and gradually get into the habit of checking in on yourself. Then you can carry that over to your studies: setting fixed times to study, testing yourself and improving bit by bit. It helps a lot with my studies, making it easier. And for daily life, it’s been good for my routine too, keeping things more regular. (P6)

Doing it (fitness self-testing) has helped me get into a really disciplined exercise routine. I’ve been keeping it up for a long time now, thanks to them. They let me see how far I can push myself with all the working out, and that gives me a good sense of validation when it comes to my fitness. (P7)

## 5 Discussion

Higher education is a critical stage for promoting HRF, as it offers one of the final large-scale opportunities to equip young people with the knowledge and habits needed for lifelong health (Scroggs et al., 2025; Zhang et al., 2024). However, studies have documented a decline in the overall health status of Chinese college students, highlighting the need for effective strategies to counter negative health trajectories (Dong et al., 2023; Zhang et al., 2024). Accordingly, structured health promotion initiatives in higher education are essential for addressing immediate health concerns and supporting lifelong physical activity and well-being (Abula et al., 2020; Bao et al., 2020; Dong et al., 2023).

As a relatively new approach to assessing HRF, fitness self-testing allows students to independently evaluate and monitor their fitness levels (Keating et al., 2020; Ryan, 2023). However, limited research has examined this practice among college students. This study addresses that gap by exploring first-year Chinese male college students’ perceptions of fitness self-testing through the framework of SDT.

Of greater importance, because the participants were exclusively male college students, their experiences may have been shaped by gendered expectations related to physical competence, performance, competitiveness, and body image in the Chinese cultural context. These factors may have influenced how students interpreted fitness self-testing and responded to peer relatedness within the study.

In contrast to findings from previous studies on traditional fitness testing (Ashley and Kawabata, 2023; Harte et al., 2025; Wrench and Garrett, 2008), the current study did not reveal sharply polarized perceptions. Instead, all three core components of SDT—autonomy, competence, and relatedness—were consistently expressed in participants’ perceptions. In line with the findings reported in the literature on the topic (Graser et al., 2011; Liu et al., 2024), the data from the current study suggest that fitness self-testing for college male students offers a balanced and supportive experience. These findings highlight the potential of fitness self-testing to serve as an effective pedagogical tool for promoting lifelong engagement in HRF activities. However, it is important to note that cultural context may influence students’ autonomy, competence, and relatedness. For example, students’ prior educational experiences in China, where annual fitness testing is required from Grades 3 through 12, along with cultural beliefs about exercise and health and social norms surrounding masculinity and physical performance, may have shaped their experience to engage in self-testing and share fitness data with instructors and peers. In addition, participants’ unique living situation—living on campus without part-time employment, which is common among Chinese university students—may have influenced their engagement with the intervention. Therefore, the findings should be interpreted within this specific gendered and cultural context, and future research should examine how students from different gender identities, cultural backgrounds, and living situations experience fitness self-testing and SDT-based motivational processes.

### 5.1 Theme 1: autonomy as empowerment and responsibility

Theme 1 suggests that while autonomy enhances engagement, it also highlights the need for scaffolding strategies that help students sustain motivation when external assistance is reduced. This result aligns with previous literature showing that empowerment requires self-discipline (Pettersson et al., 2021). While students appreciated the flexibility and reduced external pressure associated with self-testing, they also acknowledged the heightened demand for self-discipline. This tension highlights both the empowering potential of autonomy and the challenges it poses when students are required to self-regulate without external requirements. Moreover, it is important to note that the participants in this study were first-year college students who were only beginning to take responsibility for their own lives. The challenges they reported regarding self-discipline may, therefore, reflect their transitional stage of development. As students gain more experience managing independence, the perceived difficulty of maintaining self-discipline may gradually diminish, allowing them to more effectively sustain fitness self-testing practices over time.

### 5.2 Theme 2: **c**ompetence satisfaction mixed with frustration

This theme revealed that although students found the testing procedures straightforward, they often struggled to make tangible improvements in HRF because they lacked the foundational knowledge and skills needed to improve specific HRF components. This finding has not been well documented in the existing literature on fitness self-testing. Because the self-testing approach required participants to take responsibility for improving their own fitness rather than simply completing required fitness tests as they had done previously, participants experienced competence frustration during the process for the first time. This finding both aligns with and extends previous SDT research, which identifies competence as a basic psychological need and a key factor influencing motivation (Barbosa Cano and Gomez-Baya, 2025). Specifically, the findings suggest that when students recognize gaps between their current fitness levels and desired outcomes but lack the knowledge or skills to make improvements, fitness self-testing may initially produce competence frustration while also creating opportunities for deeper learning and motivational development. Moreover, it is important to note that this finding has not been previously reported in physical education research, as no empirical studies on this topic have examined students’ perceived competence in improving their own HRF. The important contribution of this finding is that it extends current physical education research by shifting attention from students’ performance on fitness tests to their perceived competence in improving their own HRF. Previous research has largely examined fitness testing as an assessment tool (Silverman et al., 2008), whereas this study highlights the need to use fitness testing as an instructional process that helps students understand their results, identify areas for improvement, and learn specific strategies for enhancing HRF. By revealing students’ competence frustration during fitness self-testing, this finding underscores the importance of teaching students not only how to complete fitness tests but also how to use test results to guide meaningful fitness improvement. This result calls for more efforts to teach HRF effectively in all educational levels, even though fitness education has been included in PE teaching standards worldwide (Scroggs et al., 2025; Harris et al., 2018; Harte et al., 2025; Yang et al., 2025). This gap between effort and perceived progress points to the importance of competence as a critical factor in sustaining motivation. This finding is also consistent with the socio-cultural approach and comparable to learning motor skills in PE (Lynch, 2024). Students require continued scaffolding and guidance from an expert to assist the student to become competent. Future research should examine how instructors can help students enhance their HRF based on their testing results.

### 5.3 Theme 3: self-testing as a socially embedded practice

This theme illustrates that relatedness plays a central role in shaping positive attitudes toward self-testing, as peer connections transformed the activity from an individual task into a socially meaningful experience. For college students, building social connections has been viewed as an essential part of their lives (Wang et al., 2024). Therefore, it is not surprising that participants in the study valued peer connections and seldomly mentioned about the relatedness with their instructor, TAs and/or family relatives. By providing opportunities for peer interaction and the formation of new friendships with the same commitment and capability, self-testing transformed what could have been an isolated task into a socially meaningful activity. This finding supports previous literature showing that relatedness enhances enjoyment and persistence in health behaviors (Belanger and Patrick, 2018; Bhuiyan et al., 2022; Van Luchene and Delens, 2021).

The limited connectedness between the instructor or TAs and the participants may reflect the combined influence of Chinese cultural norms, instructional structure, and students’ perceptions of autonomy. Specifically, students may have been less likely to seek help from the instructor or TAs because of cultural expectations related to respecting authority, avoiding embarrassment, or not wanting to appear overly dependent. At the same time, the self-testing structure may have encouraged students to rely more on peers and personal responsibility when interpreting their fitness results and planning for improvement. However, it is also possible that some students may experience isolation because self-testing allows them to complete the activities independently. As such, this finding suggests the need for more structured instructional support to help students develop stronger connections with their peers, the instructor and/or TAs. Because teaching and learning are reciprocal processes, and improving students’ HRF is a shared goal (Alfrey, 2024; Hamilton et al., 2025), intentional instructor and TA support may be essential for helping students translate fitness testing results into meaningful fitness improvement. The central expression of relatedness on peers may support the finding that first year college students prefer to make their adjustment (Shin and Adame, 2024), even when they were struggling with the improvement of their HRF. It is also surprising the connection with the PE instructor and/or TAs was not established. The absence of relatedness with PE instructors and TAs warrants further examination. One plausible explanation is that participants interpreted autonomy as requiring complete independence. As a result, they were unlikely to seek support from the PE instructors or TAs, even when they needed guidance to improve their HRF, which limited opportunities to develop relatedness with instructional staff. Similarly, parents were rarely involved in the self-testing process, considering first-year students appeared eager to demonstrate independence and self-sufficiency (Shin and Adame, 2024). More research on the topic is needed in the future.

### 5.4 Theme 4: self-testing as a catalyst for self-challenge, confidence, and self-regulated lifestyle development

This finding indicates that when autonomy, competence, and relatedness are addressed simultaneously, fitness self-testing functioned as more than a measurement activity, providing a pathway for students to internalize fitness behaviors as part of their lives. The emergence of this theme further suggests that fitness self-testing may have benefits that extend beyond physical activity participation. Through setting goals, tracking progress, and maintaining exercise routines, students developed confidence, discipline, and self-regulatory habits that were not limited to fitness contexts. As students learned to monitor their own performance, identify areas for improvement, and work toward gradual progress, they began to develop a broader sense of personal responsibility and self-management. These skills appeared to transfer to other areas of university life, including studying, research, and daily routines, suggesting that fitness self-testing may serve as a meaningful context for developing transferable self-regulation skills (Graser et al., 2011; Cao and Jiang, 2025). When students begin to view fitness testing as a tool for self-improvement rather than as an external requirement, they may be more likely to develop a sense of ownership over their health-related behaviors. This shift from compliance to personal investment is important because it may help students sustain physical activity beyond the immediate testing context. In this way, fitness self-testing can serve as a driving force for long-term engagement in fitness and support the development of lifelong health-related habits (Keating et al., 2020).

Taken together, these findings reinforce SDT’s central claim that environments supporting autonomy, competence, and relatedness may be effective in promoting sustained engagement and behavior change (Barbosa Cano and Gomez-Baya, 2025). They further suggest that fitness self-testing may move beyond assessment when it is thoughtfully integrated with instructional guidance, goal setting, and social support. In this way, fitness self-testing may serve as a powerful pedagogical tool for helping college students develop lifelong health behaviors.

## 6 Limitations

This study employed a qualitative approach to explore the perceptions of fitness self-testing among first-year male college students in China. While this design provided rich insights into students’ experiences in life, it does not allow for causal inferences. Furthermore, caution should be exercised when generalizing these findings to different populations or conceptual frameworks, as perceptions may vary across sex, cultural settings, and stages of college life. Finally, although students in the present study were afforded some control regarding when, where, and how frequently they completed fitness self-testing, all participants were enrolled in a mandatory physical education course and were required to complete self-testing once per month, with instructor-led testing conducted at the beginning and end of the semester. These structural constraints limit the extent to which students’ participation can be characterized as truly autonomous.

## 7 Implications for future research

The findings suggest that fitness self-testing can be implemented effectively in higher education physical education settings when it is embedded within a structured pedagogical process rather than used solely as an assessment activity. For example, instructors can begin by teaching students how to conduct fitness self-tests safely and accurately, interpret their results, and identify specific HRF components that need improvement. Students can then set individualized short-term goals, such as improving aerobic fitness, muscular endurance, flexibility, or exercise consistency, and complete repeated self-testing opportunities throughout the semester to monitor their progress as suggested by Keating and colleagues (Keating et al., 2020). Furthermore, Franco and colleagues (Franco et al., 2025) have recommended that instructors may also ask students to develop weekly exercise plans based on their self-testing results, maintain brief reflection logs, and discuss progress with peers to promote autonomy, competence, and relatedness. More importantly, physical education instructors should provide one-on-one meetings to help students identify effective strategies for improving their HRF as effective feedback is found to be important to the quality of student learning (Huang et al., 2026; Treschman et al., 2024). In this way, fitness self-testing can help college students connect assessment results with meaningful behavior change and develop self-regulated habits that support lifelong physical activity.

Future research should build on these findings by including more diverse samples, such as female students and those from different academic years or cultural backgrounds. Longitudinal studies would be particularly useful for examining how perceptions of fitness self-testing change over time, especially as students mature and develop stronger self-discipline. In addition, mixed methods and experimental designs could help establish causal links between fitness self-testing and outcomes such as physical activity levels, psychological need satisfaction, and overall well-being. Intervention-based research would also provide valuable evidence on the effectiveness of integrating self-testing into PE curricula, offering practical strategies to enhance autonomy, competence, and relatedness in higher education contexts. Similar to the development of a scale for measuring innovative performance evaluation among personal fitness trainers (Chankuna and Sukdee, 2025), greater attention should be given to developing a questionnaire that measures college students’ basic psychological needs in the context of fitness self-testing. Such a scale is urgently needed to support future research on this topic and to better examine how autonomy, competence, and relatedness shape students’ motivation and engagement in fitness self-testing.

## 8 Conclusion

This study provides initial qualitative evidence on Chinese male college students’ perceptions of HRF self-testing and offers new insight into how self-testing may support motivation and engagement within the framework of SDT. Four themes emerged from the data: autonomy brought flexibility and responsibility; competence involved both procedural confidence and frustration with improving HRF outcomes; relatedness developed primarily through peer interaction and support; and self-testing served as a catalyst for self-challenge and self-regulated lifestyle development. Together, these findings suggest that HRF self-testing can move beyond assessment to become a meaningful pedagogical tool that encourages students to take ownership of their fitness, monitor progress, and develop more sustainable health behaviors.

At the same time, the findings highlight the importance of structured instructional support. Although students appreciated the flexibility and personal responsibility associated with self-testing, some struggled to translate test results into effective strategies for improvement. Therefore, instructors should not assume that students can improve their HRF simply by conducting self-tests. Instead, fitness self-testing should be paired with guidance on interpreting results, setting realistic goals, developing individualized exercise plans, and seeking support from peers, instructors, or teaching assistants. Overall, this study suggests that when HRF self-testing is implemented with appropriate pedagogical support, it has the potential to promote autonomy, competence, relatedness, and long-term engagement in physical activity among college students.

## Data Availability

The raw data supporting the conclusions of this article will be made available by the authors, without undue reservation.
